# Effect of Heating/Cooling Rate and Temperature on Microstructure and Electrical Properties of Sputter-Deposited PZT Thin Films Crystallized by Conventional Furnace Annealing

**DOI:** 10.3390/ma19091782

**Published:** 2026-04-28

**Authors:** Manfred Wich, Jan Helmerich, Philipp Ott, Oliver Ambacher, Stefan Johann Rupitsch

**Affiliations:** 1 Laboratory for Electrical Instrumentation and Embedded Systems, Department of Microsystems Engineering (IMTEK), University of Freiburg, 79110 Freiburg im Breisgau, Germany; 2 Power Electronics, Institute for Sustainable Systems INATECH, University of Freiburg, 79110 Freiburg im Breisgau, Germany; 3 BrainLinks-BrainTools, 79110 Freiburg im Breisgau, Germany

**Keywords:** annealing treatment, characterization of piezoelectric materials, conventional furnace annealing (CFA), dielectric, ferroelectric thin film, lead zirconate titanate (PZT), perovskite structure, RF magnetron sputter deposition

## Abstract

Lead zirconate titanate (PZT) is a widely used material for applications in microsensors, actuators, and transducers. Due to its high piezoelectric coefficient, large dielectric constant, and strong polarization capability near the morphotropic phase boundary (Zr/Ti ≈ 52/48), it is considered one of the most attractive materials for micro-electromechanical systems (MEMS). These advantageous material properties strongly depend on the PZT layer’s microstructure and crystallinity, which are primarily determined by the choice of seed layer, deposition conditions, and the post-deposition annealing treatment that promotes the formation of the PZT’s perovskite phase. In this contribution, sputter-deposited PZT thin films were crystallized by conventional furnace annealing (CFA) to evaluate the effect of heating/cooling rates (1 °C·min^−1^–7 °C·min^−1^) within a temperature range of 450 °C to 700 °C on structural, electrical, and ferroelectric properties, with consideration of the seed layer preparation. We characterized the materials’ properties by X-ray diffraction (XRD), scanning electron microscopy (SEM), atomic force microscopy (AFM), and measurements of the ferroelectric hysteresis, capacitance, and leakage current. All samples annealed at temperatures of at least 500 °C fully crystallized into the perovskite phase, independently of the heating/cooling rate. The best ferroelectric performance was achieved at 550 °C with a 1 °C·min^−1^ heating/cooling rate, yielding a saturation polarization of 82.8 µC·cm^−2^ and a remnant polarization of 36.9 µC·cm^−2^ under a maximum applied field of 300 kV·cm^−1^.

## 1. Introduction

Lead zirconate titanate Pb(Zr_x_Ti_1−x_)O_3_ (PZT) thin films have been employed in a wide range of applications, including SAW filters, ferroelectric random access memory, electro-optic or acousto-optic waveguides and various kinds of micro-electromechanical systems (MEMS), such as magnetoelectric devices, piezoelectric ultrasonic transducers and vibration energy harvesters [1,2,3,4,5,6,7,8,9]. The material’s superior electrical properties at the morphotropic phase boundary (Zr/Ti ≈ 52/48) are the reason why PZT is regarded as one of the most important ferroelectric materials [2,10,11]. These properties comprise a large electromechanical coupling coefficient, enhanced dielectric properties, strong polarization capability, and a high Curie temperature [5,10]. However, the advantageous electrical and ferroelectric properties arise only after the PZT thin film is crystallized to a pure perovskite phase, since films are typically amorphous or contain pyrochlore phases even at elevated deposition temperatures, and therefore require a post-deposition annealing treatment [2,12]. Achieving reliable crystallization remains a challenging task in the fabrication of PZT thin films, requiring control and prevention of issues such as lead loss, film delamination, and the formation of cracks and hillocks [11,12]. The following paragraphs cover the primary factors influencing the crystallization process, which are the film fabrication method and its parameters, the selection and preparation of the seed layer, and the annealing treatment [2,13].

Radio-frequency (RF) magnetron sputtering and the sol–gel technique represent the most extensively studied methods for the preparation of PZT thin films, categorized as physical vapor deposition and chemical solution deposition, respectively [2,14,15]. The sol–gel process, in which stoichiometric precursor solutions are spin-coated onto the substrate and annealed to form solid oxide films, suffers from property-degrading deficiencies, such as low crystallinity, weak interface adhesion, poor uniformity, and residual impurities [2]. RF magnetron sputtering utilizes a plasma in which ions are accelerated by an alternating high-frequency electric field toward a target material, causing an ejection of atoms or atomic clusters from the target, which then deposit on the substrate, yielding the thin film [14]. A magnetic field increases the ionization rate of the inert gas, thereby enhancing the deposition rate [14]. Sputtering does not exhibit the aforementioned disadvantages and, moreover, yields films with more reproducible properties, excellent compositional uniformity across large areas, improved surface smoothness, and readily controllable thickness [10,12,13]. Furthermore, Shilpa et al. [15] demonstrated that the remnant polarization *P*_r_ and the coercive electric field *E*_c_ of sol–gel PZT films are lower than those of sputtered PZT films. The composition of the sputter-deposited film depends not only on the target composition but also strongly on the deposition temperature, gas atmosphere, and sputter power [2,12]. Velu et al. [16] exploited these dependencies to achieve precise control over the film composition, making this a significant advantage of sputtering. In this process, the high volatility of Pb makes the precise control of Pb stoichiometry in the deposited film essential, since lead loss facilitates the formation of the non-ferroelectric pyrochlore phase [12]. Sufficient Pb content in the film is typically ensured either by using a target with excess PbO or by sputtering onto an unheated substrate, which effectively inhibits lead evaporation during deposition and can even yield a Pb/(Zr + Ti) ratio in the deposited film that exceeds that of the target [12,17]. Hence, this contribution focuses on the sputtering technique with deposition of the thin films onto unheated substrates.

The choice of the substrate combined with the seed layer and their preparation strongly affect the PZT’s microstructure, such as grain size, orientation, stress, and crystallization during the post-deposition annealing [2,15]. The substrate should possess a lattice constant and coefficient of thermal expansion (CTE) closely matching those of PZT, making platinized silicon substrates (Si/SiO_2_/Ti/Pt) the most commonly used for microelectronic applications [2,5]. Platinum features a small lattice mismatch of only 3% (*a*_PZT_ = 4.036 Å, *a*_Pt_ = 3.924 Å), a similar coefficient of thermal expansion (CTE_PZT_ = ~11 × 10^−6^ K^−1^, CTE_Pt_ = 8.8 × 10^−6^ K^−1^), an excellent chemical stability, a relatively low crystallization temperature, a high thermal conductivity, and good electrical conductivity that enables its use as a bottom or top electrode [2,5,18,19]. While the SiO_2_ layer provides electrical insulation from the silicon substrate, the titanium film is essential for enabling interfacial bonding between Pt and SiO_2_ and therefore serves as an adhesion layer [2,5,18]. During the post-deposition annealing of PZT, the substrate’s Ti and Pt films may experience thermally driven chemical and microstructural changes, which impact the PZT film both beneficially and detrimentally [20]. These changes include the recrystallization of Pt and the diffusion of Ti along Pt grain boundaries toward the Pt/PZT interface [2,20,21,22]. The recrystallization of platinum, which alters the film’s grain sizes, and the diffusion of titanium atoms, which can oxidize to TiO_x_ within the grain boundaries, both generate significant compressive stress in the Pt layer [18,21,22,23]. Compressive stress within the Pt seed layer may lead to the formation of hillocks that substantially degrade the PZT layer, facilitate the development of cracks resulting in electrical short circuits between the top and bottom electrodes, or induce delamination of the PZT film [5,18,20,21,22,23,24,25]. Yet hillock formation can be suppressed by replacing the Ti adhesion layer with TiO_x_, which drastically reduces the Ti diffusion into the Pt layer, since the diffusion velocity of TiO_x_ is lower than that of metallic Ti [5,20,26]. However, Jeong et al. [19] reported a larger remnant polarization and better crystallinity for PZT films deposited on Ti/Pt electrodes than for those deposited on TiO_2_/Pt. Improved crystallinity results from Ti atoms that migrated to the Pt/PZT interface, where they serve as nucleation sites for the crystallization of PZT and promote the formation of the desired perovskite phase [2,19,24,27]. Following the approach in [26], we introduced an additional annealing step prior to PZT deposition to stabilize the Si/SiO_2_/Ti/Pt substrate, thereby preventing any mechanical stresses arising from the thermally driven chemical and microstructural changes from being transferred to the PZT layer. Furthermore, enhanced PZT properties are observed when the substrate is thermally treated prior to PZT deposition [18,19,22]. Consequently, this additional annealing step can improve the PZT layer’s properties by promoting Ti diffusion to form nucleation sites and by improving Pt crystallinity, which can increase the average grain size in the PZT layer [2].

PZT thin films deposited at low temperatures typically exhibit an amorphous or pyrochlore structure, while even at high deposition temperatures, the film can display a coexistence of the pyrochlore and perovskite phases [2,14]. Since only the perovskite PZT phase features ferroelectric properties, a post-deposition annealing treatment is frequently required, during which the as-deposited film transforms from an amorphous or metastable pyrochlore structure into the stable perovskite phase [2,13,14,28]. Thermal annealing treatments include conventional furnace annealing (CFA) with slow heating rates in the range of several degrees per minute (°C·min^−1^), and rapid thermal annealing (RTA) with high heating rates of multiple degrees per second (°C·s^−1^) [28]. The rapid heating rates and the short annealing times of RTA minimize the thin film’s thermal budget, thereby decreasing Pb volatilization due to interdiffusion, whereas CFA, which is commonly carried out in a muffle furnace in an air atmosphere, offers the benefit of less complex equipment requirements [2,14]. Extensive research has been devoted to comparing these annealing methods and examining their influence on the PZT microstructure, which determines the thin film’s ferro- and piezoelectric properties, with attention to thermal processing parameters such as annealing temperature, dwell time, and heating rate [2,5,16].

A sufficiently high temperature is required to improve crystallinity and ensure the full transition of the PZT film into the perovskite phase. However, the temperature must be limited to prevent Pb volatilization that promotes the formation of the pyrochlore phase, film cracking, and degradation of substrate adhesion [2,14,29]. CFA-treated, magnetron-sputtered PZT films near the morphotropic phase boundary deposited on unheated Si/SiO_2_/Ti/Pt substrates were observed to fully transform into the perovskite phase under minimum reported temperatures and dwell times of 500 °C for 120 min (Velu et al. [16]), 550 °C for 60 min (Thongrit et al. [10]), 550 °C for 30 min (Wang and Zou [13]), 560 °C for 10 min (Huang et al. [30]), 600 °C for 60 min (Hu et al. [31]) and 525 °C for 300 min, 550 °C for 45 min, and 600 °C for 30 min (Thomas et al. [32]). Yet, according to Velu et al. [16], for PZT prepared without excess Pb, annealing temperatures above merely 625 °C with dwell times exceeding 30 min led to a decrease in the intensity of the perovskite phase and the reformation of the lead-deficient pyrochlore phase. Wang and Zou [13] likewise reported a reduction in permittivity and in remnant polarization *P*_r_ when annealing temperatures reached 625 °C and 600 °C, respectively. This observed property degradation at elevated annealing temperatures reiterates the importance of controlling lead content for PZT crystallization [16]. For RTA treatments, aimed at reducing Pb volatilization, the minimal annealing conditions required to obtain a thorough perovskite phase were determined to be 450 °C for 1 s (Hu et al. [31]), 550 °C for 1 min (Velu et al. [16]), 575 °C for 1 min (Huang et al. [30]), 650 °C for 1 min (Zhu et al. [33]) and 650 °C for 10 min (Chang and Chen [29]). The comparatively higher temperature and dwell duration reported by Chang and Chen [29] may be explained by the elevated PZT deposition temperature of 350 °C, yielding a less reactive film that requires additional energy to induce the crystallographic phase transformation. Despite RTA’s rapid heating rates, the temperature should not be set too high or held unnecessarily long, as Zhu et al. [33] observed a reduction in *P*_r_ for temperatures of 675 °C and above, and Chang and Chen [29] found inferior perovskite-phase quality for excessively high temperatures (850 °C for 5 min) or prolonged annealing times (650 °C for 40 min).

From these data, it is evident that, alongside temperature, the dwell time is a key variable influencing the transformation into the perovskite phase. Hu et al. [31] reported that, using a heating rate of 10 °C·min^−1^, a dwell time of 60 min for CFA-treated PZT at 600 °C was required to fully eliminate the pyrochlore phase, whereas with no dwell time, a residual pyrochlore phase remained detectable even at 700 °C. Thomas et al. [32] also demonstrated that extended dwell times can reduce the required peak temperature for complete transformation to the perovskite phase for CFA-treated PZT. Their results show that a dwell time of 5 h at 525 °C is required to fully eliminate the pyrochlore phase, decreasing to 45 min at 550 °C and to 30 min at 600 °C. Although the film fully crystallized to the perovskite phase in all three cases, distinct polarization properties were observed, with remnant polarization increasing as temperature rose, yielding *P*_r_ = 15 µC·cm^−2^ (525 °C, 5 h), *P*_r_ = 18 µC·cm^−2^ (550 °C, 45 min) and *P*_r_ = 22 µC·cm^−2^ (600 °C, 30 min), while a further increase in temperature resulted in a slight reduction to *P*_r_ = 21 µC·cm^−2^ (650 °C, 30 min). Similar observations were made by Chang and Chen [29] for RTA-treated PZT, where an increase in dwell time from 5 min to 10 min at 650 °C was required to eliminate the pyrochlore phase, whereas a 5 min dwell time necessitated raising the temperature to above 700 °C.

The primary difference between CFA and RTA is the heating rate, which significantly influences the PZT’s nucleation process. Due to the slow heating rate, CFA-treated PZT typically crystallizes first from the amorphous phase into the pyrochlore phase and subsequently into the perovskite structure [13,14]. In contrast, RTA-treated PZT may transform directly from the amorphous state into the perovskite phase [28]. Hu et al. [31] argued that sputtered, amorphous-state PZT films deposited on unheated substrates store substantial excess energy, which can significantly promote and induce nucleation and crystallization upon release. During CFA-treatment, this excess energy is released by relaxation of the amorphous phase at temperatures too low to enable crystallization, whereas RTA’s much higher heating rate prevents sufficient time for the amorphous structure to relax in the low-temperature region. In addition, the heating rate may influence the orientation of the polycrystalline thin film. According to Du et al. [34] and Muralt [35], a (100) orientation is preferred in PZT thin films, as it features enhanced piezoelectric properties. Huang et al. [30] obtained a dominant (100) orientation for CFA-treated PZT, while RTA-treated films were highly (111)-oriented, attributing these differences to distinct nucleation mechanisms. According to the authors, nucleation during CFA is predominantly driven by defects and impurities within the film, which favor the (100) orientation owing to its low nucleation energy barrier, whereas during RTA, nucleation mainly occurs at the Pt(111)/PZT interface, which promotes the (111) orientation due to the small lattice mismatch. Mardare et al. [22] similarly observed (100) and (111) orientations for CFA- and RTA-treated PZT, respectively, but attributed this difference to varying compressive stress in the Pt layer from interdiffusing Ti, which can be reduced through higher heating rates. While other publications [10,36,37] also reported (100)-oriented films following CFA-treatment, some studies measured (100) orientation after RTA ([16,28]). However, CFA may be favored over RTA since it allows the formation of (100) orientation and also yields larger grain sizes than RTA. The latter aspect is attributed to the lower heating rate comprising an extended annealing process. Larger grains lead to superior electrical properties such as a higher dielectric constant, improved polarization characteristics, and enhanced piezoelectric properties, which are ascribed to the increased domain wall mobility associated with larger grain size [2,5,14,16,22].

Table 1 summarizes selected studies that examined the effects of annealing temperature, dwell time, and heating rate on the PZT’s microstructure and presents their investigated parameter variations. Both the annealing temperature and dwell time have been the subject of extensive study, whereas the heating rate has primarily been studied with respect to differences between CFA and RTA. Lu et al. [38] observed that, for RTA-treated PZT, the nucleation process is more sensitive to the heating rate than to the dwell time. However, to the best of the authors’ knowledge, there is a lack of publications examining the impact of different heating rates for sputter-deposited PZT on Si/SiO_2_/Ti/Pt substrates treated via CFA.

This contribution presents a detailed analysis of the influence of various CFA heating/cooling rates (1 °C·min^−1^–7 °C·min^−1^) on the structural, electrical and ferroelectric properties of RF magnetron-sputtered PZT films on Si/SiO_2_/Ti/Pt substrates at different annealing temperatures ranging from 450 °C to 700 °C, with consideration of the seed layer preparation. XRD measurements show that all films fully crystallize into the perovskite phase at temperatures of 500 °C and above, irrespective of the heating rate, while the crystallite size exhibits greater sensitivity to the heating rate than to the peak annealing temperature for temperatures below 700 °C. A strong heating rate dependence is observed for microstrain, which increases with higher heating rates, whereas the out-of-plane lattice strain derived from the (111) diffraction peak is determined nearly entirely by the annealing temperature. The highest remnant polarization occurs at low temperatures combined with low heating rates. The leakage current remains relatively low up to 600 °C but increases significantly at higher annealing temperatures. Above 600 °C, the leakage current shows strong heating/cooling rate dependence, decreasing as rates increase.

## 2. Materials and Methods

### 2.1. Sample Fabrication

We manufactured patterned samples with accessible bottom and top electrodes to enable electrical characterization of the piezoelectric layer. Unpatterned samples, in which the piezoelectric layer covered the entire sample surface, were prepared for XRD, AFM, and SEM analysis. Additional unpatterned samples were fabricated to investigate the effect of different substrate preparation procedures.

#### 2.1.1. Patterned Sample Fabrication for Measurement of the Ferroelectric Hysteresis, Capacitance and Leakage Current

Circular patterned samples featuring an electrode diameter of 560 µm were prepared on 4-inch (100) silicon wafers. All layers were deposited by RF magnetron sputtering (FHR.Star.100-PentaCo, FHR Anlagenbau GmbH, Ottendorf-Okrilla, Germany), with the specific deposition parameters for each layer summarized in Table 2. 

**Figure 1:**
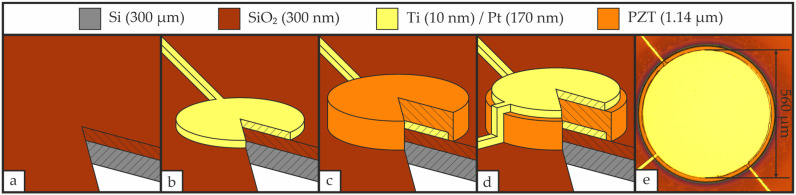
Patterned sample fabrication process. (**a**) Deposition of SiO_2_ via reactive RF magnetron sputtering; (**b**) Deposition of the Ti/Pt bottom electrode via DC magnetron sputtering and patterning via lift-off with subsequent thermal stabilization by CFA; (**c**) Deposition of PZT via RF magnetron sputtering and patterning via lift-off with subsequent crystallization by CFA; (**d**) Deposition of the Ti/Pt top electrode via DC magnetron sputtering and patterning via lift-off; (**e**) Microscopic image of a final fabricated sample.

Initially, a 300 nm SiO_2_ layer was deposited via reactive sputtering onto the cleaned wafer, providing electrical insulation (see Figure 1a). All subsequent layers were patterned by lift-off using the negative resist ma-N 1440 (micro resist technology, Berlin, Germany). We selected this resist for its 4 µm thickness and tendency to form an undercut profile during development, both of which are favorable for lift-off, as well as for its thermal stability of up to 160 °C, which is suitable for sputtering. Exposure of the photoresist was performed on an EVG 620 (EV Group, Ried im Innkreis, Upper Austria, Austria) mask aligner in soft-contact mode with an applied exposure dose of 450 mJ·cm^−2^.

A 10 nm Ti layer was sputtered onto the Si/SiO_2_ substrate, followed by 170 nm Pt, without breaking the vacuum, as seen in Figure 1b. The Ti layer serves as an adhesion layer between the SiO_2_ film and the Pt bottom electrode. Prior to the deposition of PZT, the Si/SiO_2_/Ti/Pt stack was thermally stabilized by CFA for 30 min at 700 °C, matching the maximum temperature of the subsequent PZT annealing, with a heating and cooling rate of 3 °C·min^−1^. Using a Pb(Zr_0.52_Ti_0.48_)O_3_ target, a PZT layer of 1.14 µm thickness was deposited (see Figure 1c) and subsequently annealed via CFA. In the final step, a 10 nm Ti adhesion layer and 170 nm Pt were sequentially deposited without breaking the vacuum to serve as the top electrode for electrical characterization of the PZT (see Figure 1d). All patterned layer thicknesses were verified with a Dektak 150 (Veeco Instruments Inc., Plainview, NY, USA) surface profiler.

We performed all annealing treatments by CFA (Ecotop 20, Helmut ROHDE GmbH, Prutting, Germany) in an air atmosphere and regulated temperature via a custom-built controller, monitored using a Type K thermocouple positioned in immediate proximity to the sample. The custom-built controller operates as a self-tuning, single-parameter adaptive controller, compensating for passive cooling effects at elevated temperatures. The temperature regulation is achieved via PWM control through Python (3.10.1), with the current temperature sampled every three seconds. The annealing process employed heating and cooling rates of 1 °C·min^−1^, 3 °C·min^−1^, 5 °C·min^−1^, and 7 °C·min^−1^, and peak temperatures of 550 °C, 600 °C, 650 °C, and 700 °C with a fixed dwell time of 30 min. Patterned samples were fabricated for every combination of these peak temperatures and heating/cooling rates, with the corresponding identifiers listed in Table 3.

**Figure 2:**
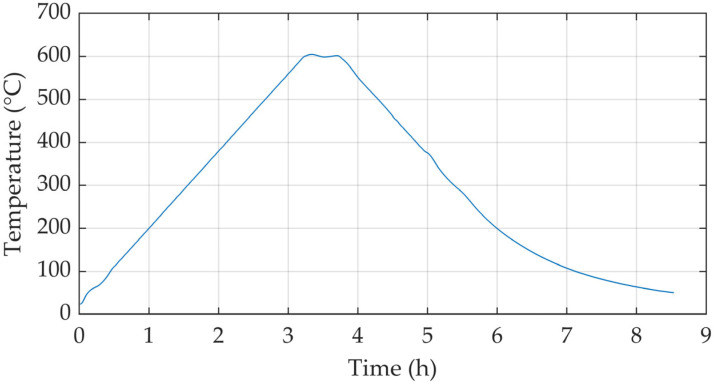
Representative temperature trace of the CFA-treatment with a heating/cooling rate of 3 °C·min^−1^, a peak temperature of 600 °C, and a dwell time of 30 min.

The temperature controller was optimized to yield a nearly linear heating rate and to avoid any significant overshoot when the peak temperature was reached. During cooling, the rate was maintained at a near-constant value before gradually transitioning into an exponential decay. Figure 2 displays a representative temperature trace measured for a heating/cooling rate of 3 °C·min^−1^ reaching a peak temperature of 600 °C.

#### 2.1.2. Unpatterned Sample Fabrication for XRD, AFM and SEM Analyses

The sample (identifier S_U_) preparation was identical to that described in Section 2.1.1, except for the Ti and Pt bottom electrode layers and the PZT film, which remained unpatterned and covered the full area of each sample. The top electrode was deliberately omitted to allow unobstructed characterization of the PZT film. Since we do not expect the deposition of a subsequent metallic top electrode to significantly affect the structural and electrical properties of the PZT film, the results obtained from unpatterned samples are considered applicable to the patterned samples, which incorporate a metallic top electrode. All unpatterned samples were fabricated on a single wafer and diced into 1.5 cm × 1.5 cm rectangular specimens prior to CFA-treatment of the PZT layer. Each unpatterned sample underwent annealing concurrently with the corresponding patterned sample in a single run, ensuring identical annealing conditions between the sample types. Besides the variations listed in Table 3, two additional samples were produced at peak temperatures of 500 °C (S_U__500_3) and 450 °C (S_U__450_3), both subjected to a heating/cooling rate of 3 °C·min^−1^.

#### 2.1.3. Sample Fabrication for Evaluating the Influence of Substrate Preparation on Hillock Formation and Delamination

We fabricated rectangular samples, similar to those in Section 2.1.2, with SiO_2_ deposited either by reactive sputtering (see Table 2) or by plasma-enhanced chemical vapor deposition (PECVD) to examine the effect of varying deposition techniques on hillock formation in the Ti/Pt bottom electrode. PECVD (STS 310PC, Surface Technology Systems PLC, Newport, UK) was performed at a temperature of 300 °C, a pressure of 900 mTorr, and a power of 30 W at 13.56 MHz. Following SiO_2_ deposition, Ti/Pt layers were sputter-deposited (see Table 2) on the entire sample’s surface and subsequently annealed by CFA at 700 °C for 30 min with a heating/cooling rate of 3 °C·min^−1^. The PZT deposition step was omitted for these samples.

An additional rectangular sample was prepared to investigate the effects of pre-annealing the Ti/Pt bottom electrode prior to the deposition of PZT on hillock formation, crack initiation, and PZT film delamination during the CFA-treatment of PZT. This sample was fabricated according to Section 2.1.2, but with the pre-annealing step omitted, to enable direct comparison with the samples that underwent pre-annealing. This sample featured a PZT layer, which, following PZT deposition, underwent CFA-treatment at 600 °C for 30 min with a heating/cooling rate of 3 °C·min^−1^.

### 2.2. Measurement Methods

We examined the microstructure of the thin films by scanning electron microscopy (SEM), atomic force microscopy (AFM), and X-ray diffraction (XRD). The electrical properties were determined by measurements of the ferroelectric hysteresis, capacitance, and leakage current.

#### 2.2.1. Scanning Electron Microscopy

SEM (XL40, Philips Electron Optics, Hillsboro, OR, USA) was used to examine the surface morphology of the Ti/Pt bottom electrode and the PZT film, while the PZT was platinized for imaging.

#### 2.2.2. Atomic Force Microscopy

Surface roughness of the PZT film was determined using an AFM (nGauge, ICSPI, Kitchener Ontario, ON, Canada).

#### 2.2.3. Microscopic Imaging

Microscopic images of the samples were captured using an optical microscope (Axioskop 2 Mat, Carl Zeiss AG, Oberkochen, Germany) and a microscopy camera (ProS5 Lite, Motic Deutschland GmbH, Wetzlar, Germany).

#### 2.2.4. X-Ray Diffraction

The present crystalline phases, crystallite size, preferential orientation, microstrain, and out-of-plane lattice strain within the PZT layer were investigated by XRD (X’Pert^3^ MRD, Malvern Panalytical, Malvern, UK) using Cu Kα_1_ radiation with a wavelength *λ* = 1.5406 Å. Measurements were performed in symmetric Bragg–Brentano geometry, maintaining *ω* = *θ* throughout the scan, with *ω* denoting the angle of incidence and *θ* the Bragg angle. The data were collected by 2θ-scans ranging from 20° to 60° with a 0.01° step increment and a counting time of 1.25 s per step. For each sample, ten measurements spanning the entire 2θ range were performed and subsequently averaged.

The average crystallite size *D* was calculated using both the Scherrer and Williamson-Hall methods. The Scherrer equation is the simplest and most widely used approach to calculate *D*, which is determined by

(1) $$D=\frac{K\lambda }{\beta \cdot cos(\theta )}$$

for each individual peak [39,40,41,42]. The Scherrer constant *K* = 0.9 is a dimensionless geometric factor, *β* denotes the corrected full width at half maximum (FWHM), and *θ* represents the Bragg angle of the observed diffraction peak. The corrected FWHM (*β*) was derived from the measured FWHM (*β*_sample_) and the instrumental FWHM (*β*_instrument_) using the relation [41]

(2) $$\beta =\sqrt{\beta_{sample}^{2}-\beta_{instrument}^{2}},$$

where *β*_instrument_ = 0.05°, which corresponds to the FWHM of the single-crystal silicon substrate, as suggested by Harrington and Santiso [39]. A limitation of the Scherrer method is that peak broadening is attributed solely to crystallite size, whereas, in addition to crystallite size, a heterogeneous distribution of strain within the crystallites (microstrain) also contributes to peak broadening [39,40,41]. The Williamson–Hall method provides a more accurate crystallite-size estimate by decomposing the FWHM of diffraction peaks in polycrystalline samples into separate contributions from crystallite size and microstrain [41]. The most commonly used form of the Williamson–Hall method is the uniform deformation model, which yields

(3) $$\beta \cdot \cos\left(\theta \right)=\frac{K\lambda }{D}+4\varepsilon \cdot \sin\left(\theta \right),$$

where *ε* is the microstrain [39,40,41]. By plotting $\beta \cdot \cos\left(\theta \right)$ against $\sin\left(\theta \right)$ for all diffraction peaks, the data are expected to follow a linear dependence [39,40,41]. The slope of this line equals 4*ε*, while the ordinate intercept equals *Kλ/D*, from which the microstrain and the crystallite size can be calculated, respectively [39,40,41]. A linear least-squares fit to the data was used to determine the slope and the ordinate intercept.

The degree of orientation *O_hkl_* for crystallographic orientation (*hkl*) in % was calculated using

(4) $$O_{hkl}=\frac{I_{hkl}/I_{hkl}^{C}}{\sum I_{hkl}/I_{hkl}^{C}}\cdot 100,$$

where *I_hkl_* is the intensity of the peak measured from the XRD pattern and $I_{hkl}^{C}$ is the standard intensity of each peak taken from the ICDD powder diffraction file for Pb(Zr_0.52_Ti_0.48_)O_3_ with $I_{100}^{C}=12$, $I_{110}^{C}=100$ and $I_{111}^{C}=15$ [22,40].

The out-of-plane lattice strain was estimated from the shift in the PZT’s (111) diffraction peak using Bragg’s law

(5) $$2d_{hkl}\cdot \sin\left(\theta \right)=n\lambda ,$$

which relates the diffraction peak’s angle of the order *n* to the interplanar spacing *d_hkl_* of the (*hkl*) plane [39,40,42]. The out-of-plane lattice strain *e*_⊥,(111)_ derived from the (111) diffraction peak was obtained using

(6) $$e_{⊥,(111)}=\frac{d_{111}-d_{111}^{C}}{d_{111}^{C}},$$

by relating the calculated interplanar spacing *d*_111_ of the (111) planes (Equation (5)) with the ICDD card reference value $d_{111}^{C}=2.351\;Å$. For tetragonal PZT, this quantity reflects the spacing of the (111) planes parallel to the thin film surface and is not identical to the c-axis out-of-plane strain.

#### 2.2.5. Ferroelectric Hysteresis

The ferroelectric hysteresis loop determines the remnant polarization *P*_r_ and the saturation polarization *P*_sat_, and was measured five times for each sample using a Sawyer-Tower circuit at a frequency of 5 kHz and a maximum applied electric field of 300 kV·cm^−1^. *P*_r_ and *P*_sat_ represent the mean values of these five individual measurements, with *P*_r_ defined as

(7) $$P_{r}=\frac{1}{2}\left(\left|P_{r,p}\right|+\left|P_{r,n}\right|\right),$$

where *P*_r,p_ and *P*_r,n_ correspond to the absolute values of the remnant polarization at zero applied electric field in the positive and negative branches of the hysteresis, respectively. A function generator (33120A, Hewlett-Packard, Palo Alto, CA, USA) and a 4-quadrant power supply (TOE7621, TOELLNER Electronic Instrumente GmbH, Herdecke, Germany) were used for the excitation, generating a sinusoidal signal, while the hysteresis loops were recorded with an oscilloscope (RTB2004, Rohde&Schwarz GmbH & Co. KG, München, Germany).

#### 2.2.6. Capacitance

The capacitance *C* was measured using an LCR meter at a frequency of 10 kHz under an applied voltage of 1 V (8.77 kV·cm^−1^). From the measured capacitance, the relative permittivity *ε*_r_ was calculated via the relation

(8) $$\varepsilon_{r}=\frac{C\cdot d}{\varepsilon_{0}\cdot A},$$

where *d* is the PZT thickness, *ε*_0_ is the vacuum permittivity, and *A* is the electrode area [1].

#### 2.2.7. Leakage Current

The leakage current was measured with a current amplifier (428 Current Amplifier, Keithley Instruments, Cleveland, OH, USA) and an oscilloscope (RTB2004, Rohde&Schwarz GmbH & Co. KG, München, Germany) while the sample was driven by a staircase-like signal provided by a function generator (33120A, Hewlett-Packard, Palo Alto, CA, USA) and amplified by a 4-quadrant power supply (TOE7621, TOELLNER Electronic Instrumente GmbH, Herdecke, Germany). The applied electric field was swept bidirectionally up to ±200 kV·cm^−1^ in 20 equidistant steps per polarity, with a 1 s stabilization period at each step. For each step, the leakage current was determined by averaging the current measured during the last 100 ms of the stabilization period.

## 3. Results and Discussion

### 3.1. Influence of Substrate Preparation on Hillock Formation and Delamination

**Figure 3:**
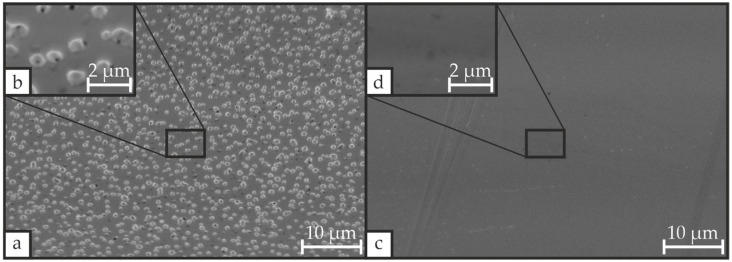
SEM images of Si/SiO_2_/Ti/Pt stacks after CFA-treatment at 700 °C for 30 min and a heating/cooling rate of 3 °C·min^−1^, where the SiO_2_ layer was deposited either by (**a**) PECVD or (**c**) reactive RF magnetron sputtering, with enlarged views (**b**,**d**) obtained from supplementary images at higher magnification.

The thermal treatment of PZT deposited on Si/SiO_2_/Ti/Pt substrate can induce the formation of cracks and hillocks that cause electrical short circuits in the PZT layer and may even cause its delamination [19,21,22,23,25,36,38,43,44,45]. The formation of such defects is strongly dependent on the substrate and its preparation. Figure 3 presents SEM images of the surface morphology of the Pt bottom electrode after CFA-treatment at 700 °C for 30 min with a heating/cooling rate of 3 °C·min^−1^ for two different SiO_2_ deposition methods.

While the Pt layer in the stack containing SiO_2_ deposited by PECVD is densely covered with hillocks, the Pt layer in the stack with sputtered SiO_2_ is free of hillocks. The observed discrepancy may be attributed to variations in oxygen diffusivity and oxygen activation energy of the two SiO_2_ variants. Susa et al. [46] reported a 42% higher diffusivity and a 48% lower activation energy for SiO_2_ deposited by chemical vapor deposition compared with sputtered SiO_2_. The presence of this excess oxygen may oxidize Ti, yielding TiO_x_, and thereby generate compressive stress that drives hillock formation. For this reason, the SiO_2_ layer for all other samples was deposited by RF magnetron sputtering.

**Figure 4:**
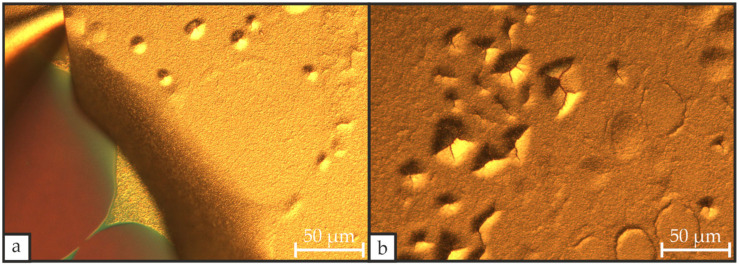
Microscopic images of a Si/SiO_2_/Ti/Pt/PZT stack after CFA-treatment at 600 °C for 30 min with a heating/cooling rate of 3 °C·min^−1^, where the Ti/Pt bottom electrode was not subjected to a separate thermal stabilization step prior to the deposition of the PZT layer, showing delamination (**a**) and hillock formation (**b**) in the PZT layer.

Figure 4 illustrates the significance of thermally stabilizing the Ti/Pt bottom electrode prior to PZT deposition. Microscopic images of the PZT layer after CFA-treatment at 600 °C for 30 min with a heating/cooling rate of 3 °C·min^−1^ depict pronounced hillock formation with associated cracking that can culminate in delamination. We were able to substantially reduce these defects by the introduction of an additional CFA-treatment of the Ti/Pt layer at 700 °C for 30 min with a heating/cooling rate of 3 °C·min^−1^, similar to the approach of Haccart et al. [26], but without replacing the Ti layer with a TiO_x_ layer, which can be beneficial to the PZT’s properties. Owing to the higher diffusivity of metallic Ti relative to TiO_x_, Ti can diffuse to the Pt surface and serve as nucleation sites, thereby promoting PZT layer crystallinity [24,27].

**Figure 5:**
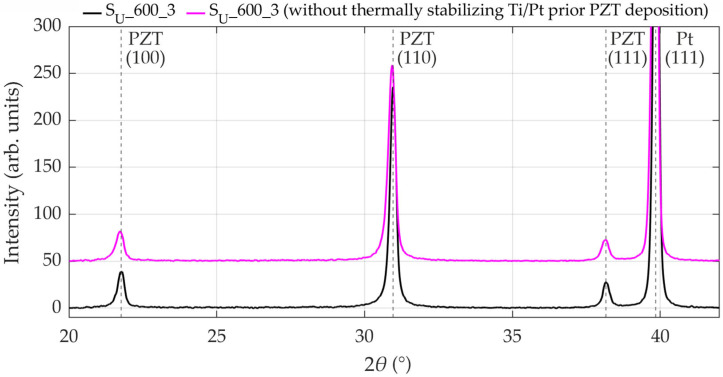
XRD patterns of two Si/SiO_2_/Ti/Pt/PZT stacks after CFA-treatment at 600 °C for 30 min with a heating/cooling rate of 3 °C·min^−1^, showing differences in PZT crystalline quality attributable to the additional thermal stabilization of the Ti/Pt layer in one sample and its omission in the other sample.

Furthermore, thermally treating the Ti/Pt layer prior to PZT deposition can increase the crystallinity of the Pt layer, which can improve the crystalline quality and the ferroelectric properties of the PZT layer, as reported by Koochekzadeh et al. [18], Jeong et al. [19], and Mardare et al. [22]. Figure 5 presents XRD patterns comparing a sample with a thermally stabilized Ti/Pt layer to a sample with an untreated Ti/Pt layer, also indicating these differences in the crystalline quality of the PZT layer. The thermally stabilized sample exhibits on average a 15% and 10% larger crystallite size according to the Scherrer method (Equation (1)) and the Williamson-Hall method (Equation (3)), respectively. The diffraction peak intensities of the thermally stabilized sample are on average 18% higher.

### 3.2. Influence of Varying Heating/Cooling Rates and Temperatures During CFA-Treatment on the PZT’s Microstructure

#### 3.2.1. Microscopic Imaging

**Figure 6:**
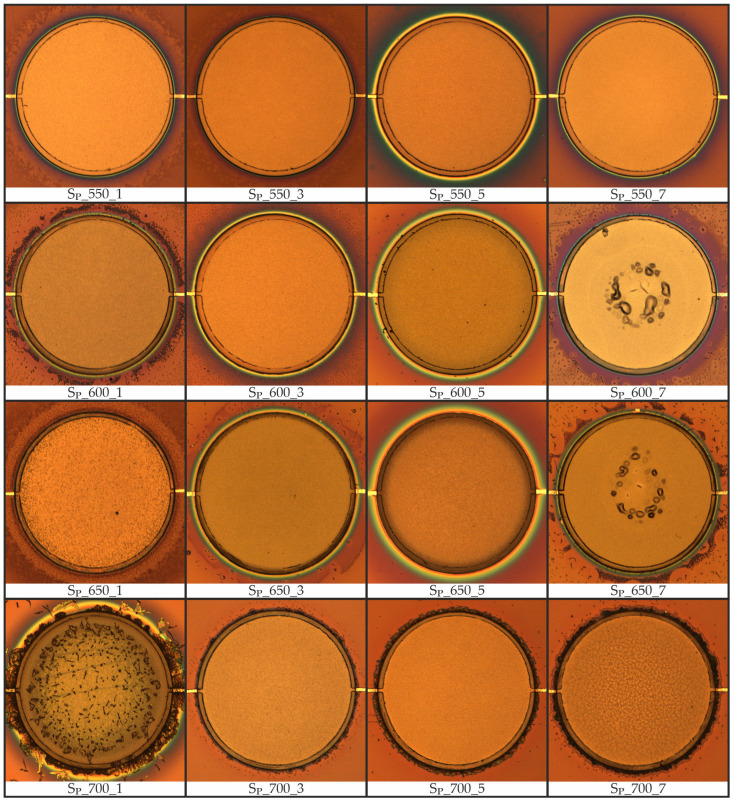
Microscopic images of the Si/SiO_2_/Ti/Pt/PZT stacks following CFA-treatment of the PZT layer prior to deposition of the top electrode for all patterned samples. Sample identifiers denote the annealing peak temperature and heating/cooling rate (e.g., S_P__650_3: 650 °C, 3 °C·min^−1^). The diameter of the patterned PZT thin film of all depicted samples is 600 µm.

The microscopic images presented in Figure 6 indicate that the surface morphology of the PZT layer is strongly influenced by the heating/cooling rate and the peak temperature applied during the CFA-treatment for PZT crystallization. For all samples that were not subjected to an excessive thermal budget or to excessively high heating/cooling rates, the PZT layer shows a smooth, homogeneous surface morphology.

Particularly under elevated temperatures combined with slow heating/cooling rates, the surface structure of the PZT thin film degrades substantially, with sample S_P__700_1 displaying the most pronounced effects. The high thermal budget during CFA-treatment under this combination is assumed to be responsible for this degradation. Chang and Chen [29], Ma et al. [2], and Zhao et al. [14] similarly report that PZT subjected to excessively high temperatures or prolonged durations tends to crack, develop pores, exhibit reduced density, and weaken substrate bonding. Despite the visual surface degradation, sample S_P__700_1 retained electrical insulation between the top and bottom electrodes after Ti/Pt top electrode deposition, indicating the absence of deep microcracks penetrating the entire PZT thickness.

Additional defects in the PZT layer observed in samples S_P__600_7 and S_P__650_7 were identified as hillocks. These hillocks, similar to those shown in Figure 4b, contain cracks that result in short circuits between the bottom electrode and the subsequently deposited top electrode. Both samples share a comparatively high heating/cooling rate of 7 °C·min^−1^ applied during the CFA-treatment of the PZT layer. This increased heating/cooling rate appears to be the cause of these defects, which is consistent with Velu et al. [16], who observed PZT layer cracking for cooling rates above 1 °C·min^−1^. Consequently, electrical shorting prevented measurement of the ferroelectric hysteresis loop, capacitance, and leakage current for both samples.

In addition to structural changes on the surface of the PZT layer, a halo-like region on the silicon surrounding the sample is observed to be strongly influenced by the thermal budget during CFA-treatment. This halo-like phenomenon does not appear to significantly affect the electrical or ferroelectric properties of the PZT layer, regardless of its extent.

#### 3.2.2. SEM

**Figure 7:**
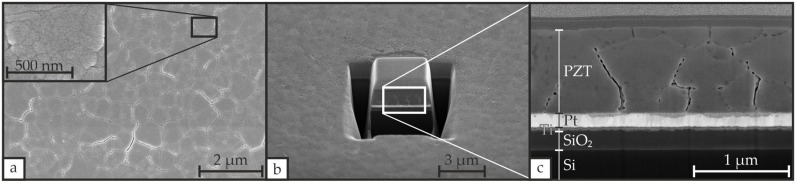
SEM images of the Si/SiO_2_/Ti/Pt/PZT stack for sample S_U__650_3 following CFA-treatment of the PZT layer, including (**a**) a top view image with visible grains and grain clusters, (**b**) a cross-sectional image showing the individual layers of the stack, and (**c**) an additional image of this cross-section captured with a higher magnification. The PZT surface was coated with platinum to facilitate SEM imaging.

Figure 7 exemplarily presents the surface and cross-sectional morphology of sample S_U__650_3 after CFA-treatment of the PZT layer at 650 °C with a heating/cooling rate of 3 °C·min^−1^. The surface morphology in Figure 7a appears homogeneous with visible grains in the sub-100 nm range and clusters of grains on the order of up to 1 µm separated by small gaps. These clusters of grains were also reported by Singh et al. [12] and exhibit similar sizes to the structures documented by Thongrit et al. [10] and Zhu et al. [47].

The cross sections in Figure 7b,c display the multilayer stack with sharp interfaces at the Si/SiO_2_, SiO_2_/Ti, and Ti/Pt boundaries and a clearly defined Pt/PZT interface, although an intermediate phase resulting from interdiffusion of the PZT layer into the Pt layer is observed. The thicknesses of the individual layers agree with the profilometer measurements summarized in Table 2. The Pt layer exhibits visible columnar grain growth, indicating highly oriented Pt, consistent with XRD measurements revealing a strong (111) orientation. Pores and voids are present in the PZT layer, as likewise reported by Ma et al. [2] and Zhu et al. [47], who attributed them to the high thermal budget or low heating rates during CFA-treatment.

#### 3.2.3. AFM

**Figure 8:**
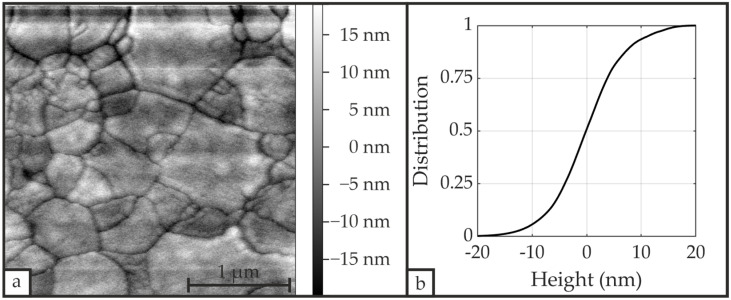
(**a**) AFM image (3 µm × 3 µm) of the PZT surface following CFA-treatment for sample S_U__650_3, exhibiting a mean roughness *R*_a_ = 6.07 nm and a root mean square roughness *R*_q_ = 7.76 nm, with (**b**) the corresponding height distribution.

The AFM image in Figure 8a depicts the surface morphology of the same sample that was examined by SEM in Section 3.2.2 and reveals structures with dimensions up to 1 µm. These dimensions match the clusters of grains identified by SEM, indicating that the observed structures likewise comprise grain clusters. The sizes of visible grains across all samples are in the approximate range from 150 nm to 300 nm. The PZT layer exhibits an arithmetical mean roughness *R*_a_ of 6.07 nm and a root mean square roughness *R*_q_ of 7.76 nm across the scanned 3 µm × 3 µm area. *R*_a_ and *R*_q_ exhibit no significant difference, which suggests an approximately symmetric surface height distribution without pronounced peaks or valleys and, consequently, a uniform surface roughness, as shown in Figure 8b. Measurements taken at multiple locations across the same sample demonstrated a high degree of surface roughness homogeneity.

**Figure 9:**
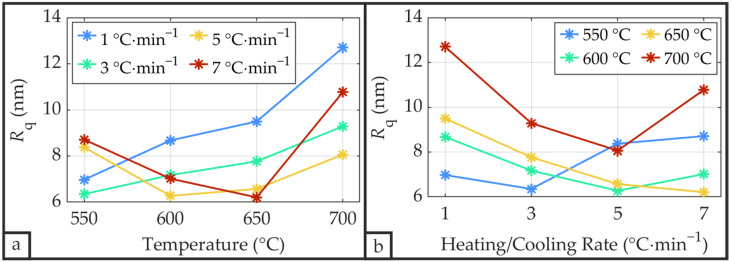
Root mean square roughness *R*_q_ of the PZT surface following CFA-treatment as a function of annealing temperature (**a**) and heating/cooling rate (**b**).

Figure 9 depicts the root mean square surface roughness *R*_q_, with an average value of 8.15 nm across all samples. The arithmetic mean roughness *R*_a_ exhibits an average value of 6.29 nm, yielding a constant *R*_a_/*R*_q_ ratio of approximately 0.75 across all samples. As shown in Figure 9, surface roughness tends to increase with increasing annealing temperature, while a slight tendency toward decreasing roughness is observed with increasing heating/cooling rate.

The measured surface roughness parameters fall within the range of values documented in previous studies. An *R*_q_ value of 33.18 nm was reported by Koochekzadeh et al. [36] for PZT films fabricated under nearly identical conditions, involving sputtering PZT onto a Si/SiO_2_/Ti/Pt substrate, pre-annealing the Ti/Pt layer at 650 °C for 30 min, and CFA-treatment of the PZT layer at 650 °C for 30 min with a heating rate of 3 °C·min^−1^. Thongrit et al. [10] and Huang et al. [30] documented *R*_q_ values of 3.83 nm and 11.6 nm, respectively, while Yan et al. [48] measured *R*_a_ values of 4 nm to 6 nm, with each study utilizing fabrication processes similar to those in this contribution.

#### 3.2.4. XRD Measurements

**Figure 10:**
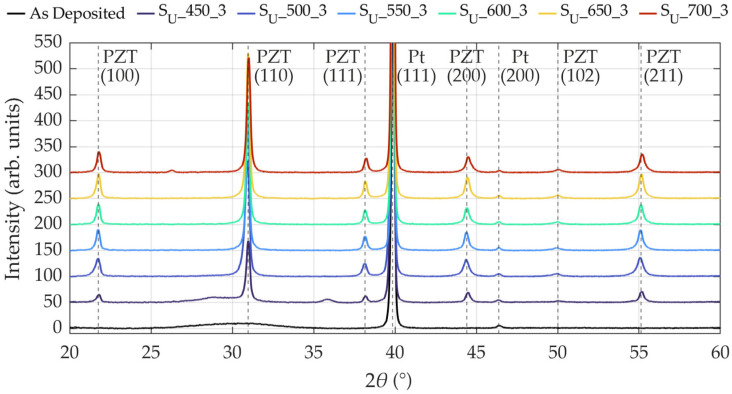
XRD patterns of Si/SiO_2_/Ti/Pt/PZT stacks with the PZT layer in the as-deposited state and after CFA-treatment at six different temperatures between 450 °C and 700 °C for 30 min with a heating/cooling rate of 3 °C·min^−1^. The Ti/Pt bottom electrode was thermally stabilized prior to PZT deposition.

The XRD pattern of the as-deposited PZT film in Figure 10 contains no characteristic peaks corresponding to the perovskite structure but reveals an amorphous morphology with only a slight intensity increase around 30°, indicating the presence of a minor pyrochlore phase [12]. Thermal stabilization of the bottom electrode prior to PZT deposition resulted in crystallization of the Pt layer, which is evident by the pronounced peak at 39.9° attributed to the (111) orientation, thereby enabling the Pt film to serve as a seed layer for the PZT perovskite phase. The subsequent thermal treatment during PZT annealing did not significantly alter the intensity or FWHM of the Pt (111) diffraction peak, confirming complete crystallization of the Pt layer prior to PZT annealing.

In addition to the as-deposited state, Figure 10 exemplarily presents the XRD patterns of samples annealed at various temperatures ranging from 450 °C to 700 °C, all of which were processed at a heating/cooling rate of 3 °C·min^−1^. For clarity and comparability to previous studies [12,16,19,20,21,22,25,29,32,33,37], overlapping diffraction peaks are represented in this contribution by a single orientation only. Accordingly, (100) refers to the combined (001)/(100) diffraction peak, (110) to (101)/(110), as it also applies to the following. Sample S_U__450_3 was annealed at the lowest temperature and already exhibits identifiable yet weakly pronounced diffraction peaks attributable to the PZT perovskite phase, while the pyrochlore phase remains present. At an annealing temperature of 500 °C, the pyrochlore phase disappears, and complete crystallization into the perovskite phase occurs. However, the diffraction peaks demonstrate relatively high FWHM values of 0.35°, 0.32°, and 0.33° for the (100), (110), and (111) orientations, respectively. The XRD patterns of all samples subjected to annealing temperatures ranging from 550 °C to 700 °C confirm that complete perovskite phase formation is maintained, showing improved crystallinity with maximum FWHM values of 0.29°, 0.26°, and 0.27° for the (100), (110), and (111) orientations, respectively. These values are comparable to those reported by Toyama et al. [45], who measured approximately 0.28° for the (100) orientation and 0.24° to 0.3° for the (110) orientation for PZT films sputtered on unheated Si/SiO_2_/Ti/Pt substrates and subsequently annealed via CFA. The observed transition from incomplete to complete perovskite phase formation in the temperature range from 500 °C to 550 °C, accompanied by improved crystallinity, aligns with literature values for CFA-treated PZT, including 500 °C for 120 min by Velu et al. [16], 550 °C for 30 min by Wang and Zou [13], and 550 °C for 60 min by Thongrit et al. [10].

**Figure 11:**
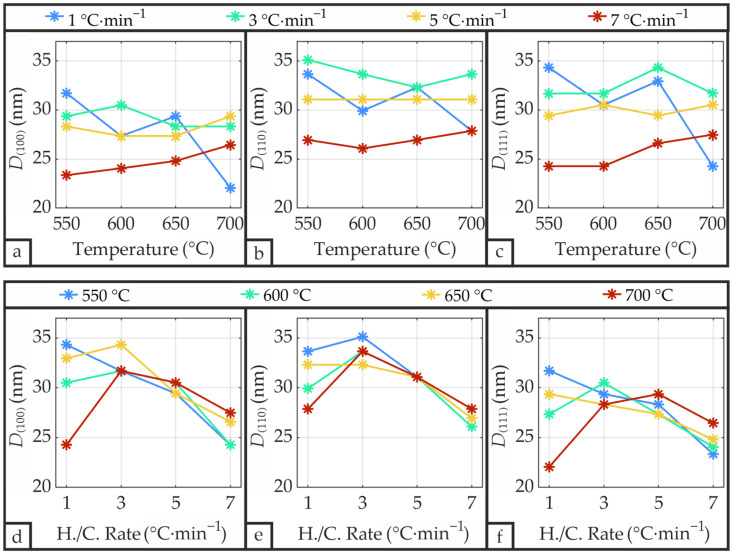
Scherrer-derived crystallite size *D* of the PZT thin film as a function of annealing temperature and heating/cooling rate calculated from the FWHM of the (100), (110), and (111) diffraction peaks using Equation (1). (**a**,**d**) Obtained from the (100) diffraction peak; (**b**,**e**) Obtained from the (110) diffraction peak; (**c**,**f**) Obtained from the (111) diffraction peak.

The crystallite size *D*, which is inversely proportional to the FWHM, was calculated from the PZT diffraction peaks of the (100), (110), and (111) orientations according to the Scherrer Equation (1) and is depicted in Figure 11 as a function of temperature and heating/cooling rate. The average crystallite size was determined to be 29.2 nm, which is comparable to the value of 24 nm reported by Natali et al. [49] for sputtered PZT on unheated Si/SiO_2_/Ti/Pt substrates and CFA-treated at 650 °C, and to the 34 nm reported by Navakoti et al. [50] for PZT prepared by conventional solid-state synthesis, yet larger than the 14.4 nm reported by Ozden et al. [51] for PZT fabricated through combined spin-coating and sputtering methods.

The Scherrer-derived crystallite size *D* remains relatively independent of temperature for heating/cooling rates of 3 °C·min^−1^ and 5 °C·min^−1^ (Figure 11a–c). In contrast, the 1 °C·min^−1^ curve exhibits a decreasing trend with increasing temperature, while the 7 °C·min^−1^ curve displays comparatively low values across all temperatures that increase slightly with temperature. The reduced crystallite sizes observed for samples annealed at 7 °C·min^−1^ may be attributed to the lower thermal budget resulting from the rapid heating and cooling phases. Conversely, the extended thermal budget encountered by samples processed at 1 °C·min^−1^ facilitates lead volatilization, especially at elevated annealing temperatures, thereby degrading perovskite crystalline quality. Apart from the exception for 1 °C·min^−1^ at elevated temperatures, Figure 11d–f demonstrate a clear decrease in crystallite size with increasing heating/cooling rate. Overall, the Scherrer-derived crystallite size reaches its maximum at low heating/cooling rates combined with temperatures sufficient for complete crystallization while minimizing lead volatilization, with 550 °C and 1 °C·min^−1^ yielding the largest average crystallite size of 33.2 nm.

**Figure 12:**
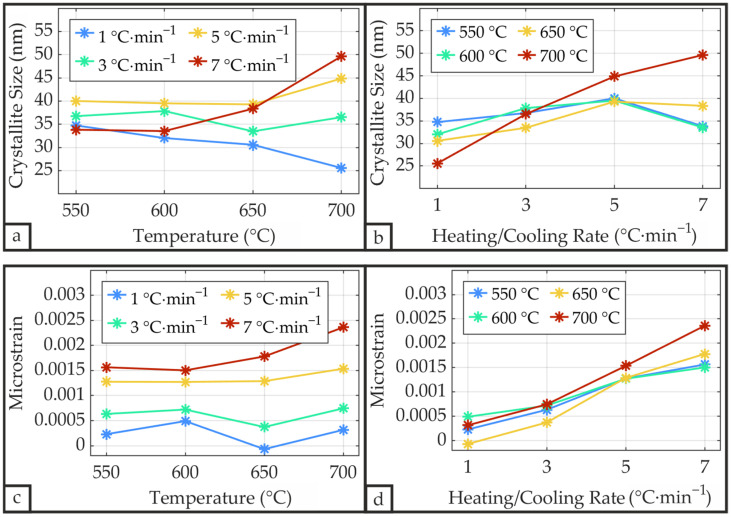
Crystallite size *D* (**a**,**b**) and microstrain *ε* (**c**,**d**) of the PZT thin film as a function of annealing temperature and heating/cooling rate determined through the Williamson-Hall method according to Equation (3).

The Williamson-Hall method deconvolves the XRD peak broadening into contributions from crystallite size and microstrain. Its analysis is depicted in Figure 12. The average microstrain of 0.0010 is comparable to the value of 0.0015 reported by Navakoti et al. [50] and of a similar order of magnitude to the values reported by Omran et al. [52] for PZT annealed between 550 °C and 700 °C, which averages 0.0039. The calculated microstrain presented in Figure 12c remains relatively independent of the temperature, apart from the 7 °C·min^−1^ curve, which shows a slightly increasing trend with rising temperature. However, the microstrain exhibits a strong dependence on the heating/cooling rate and increases substantially with increasing rate, as displayed in Figure 12d. Consequently, the contribution of microstrain to peak broadening increases with rising heating/cooling rate, resulting in an underestimation of crystallite size by the Scherrer model at elevated rates. The increasing trend in crystallite size with rising heating/cooling rate in Figure 12b can thus be attributed to this underestimation, whereas the Scherrer-derived data in Figure 11d–f show the opposite trend. Only at the maximum heating/cooling rate of 7 °C·min^−1^ does this increasing trend deviate, with samples processed at 550 °C and 600 °C showing a moderate crystallite size reduction, and the sample treated at 650 °C showing a small reduction. The crystallite size as a function of temperature in Figure 12a exhibits behavior similar to that observed with the Scherrer method, with the 3 °C·min^−1^ and 5 °C·min^−1^ rates showing minimal temperature dependence, the 1 °C·min^−1^ rate decreasing with rising temperature, and the 7 °C·min^−1^ rate increasing at elevated temperatures. Overall, the Williamson-Hall method yielded slightly higher crystallite sizes by correcting for microstrain contributions at elevated heating/cooling rates, with a maximum value of 49.6 nm for the sample annealed at 700 °C at 7 °C·min^−1^.

**Figure 13:**
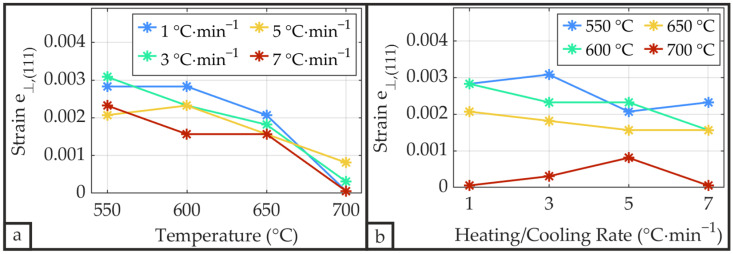
Out-of-plane strain *e*_⊥,(111)_ derived from the (111) diffraction peak of the PZT thin film as a function of annealing temperature (**a**) and heating/cooling rate (**b**), calculated using Equation (6).

The out-of-plane lattice strain *e*_⊥,(111)_ derived from the (111) diffraction peak was calculated using Equation (6) by comparing the measured interplanar spacing with the reference value obtained from the corresponding ICDD card. As shown in Figure 13a, an increase in annealing temperature results in progressively lower *e*_⊥,(111)_ values, independent of the heating/cooling rate. Similar relaxation was reported by Sreenivas et al. [53], who demonstrated that such relaxation during processing necessitates a robust film-substrate interfacial bond to prevent microcracking or delamination. Since none of the samples exhibited annealing temperature-induced microcracking or delamination, the film-substrate interfacial bonding proved to be sufficiently robust. The magnitude of *e*_⊥,(111)_ is comparable to those values reported by Sreenivas et al. [53] for a pseudocubic system and similarly approaches zero at annealing temperatures of 700 °C. Aside from a slight decrease at annealing temperatures up to 650 °C, *e*_⊥,(111)_ remains relatively independent of the heating/cooling rate, as demonstrated in Figure 13b.

**Figure 14:**
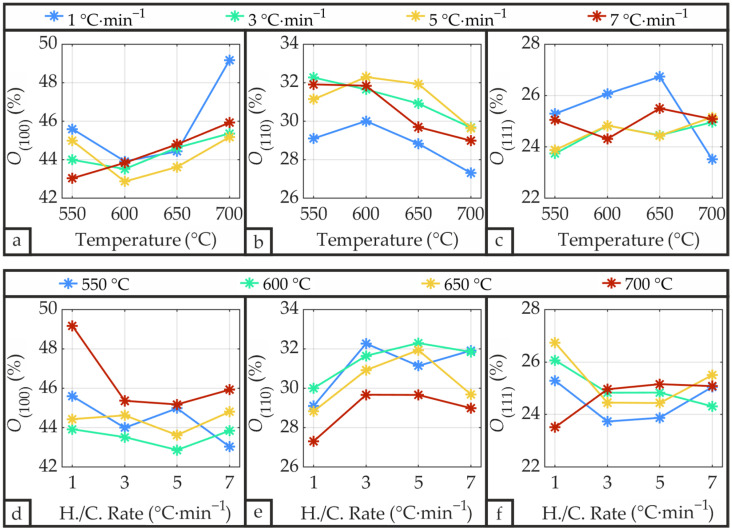
Degree of orientation *O* of the PZT thin films as a function of annealing temperature (**a**–**c**) and heating/cooling rate (**d**–**f**), calculated using Equation (4).

Figure 14 illustrates the degree of orientation *O* for the three crystallographic orientations (100), (110), and (111) of the PZT as a function of annealing temperature and heating/cooling rate.

Across all samples, the (100), (110), and (111) orientations averaged 44.7%, 30.4%, and 24.9%, respectively, with no substantial dependence on annealing temperature or heating/cooling rate. Mardare et al. [22] reported similar values of approximately 54%, 20%, and 26% for the (100), (110), and (111) orientations, respectively, for sputtered and CFA-treated PZT on Si/SiO_2_/Ti/Pt substrate, where the Pt layer was deposited at 700 °C. Overall, the predominance of (100)-oriented PZT is advantageous, as this orientation has been shown by both Du et al. [34] and Muralt [35] to exhibit superior piezoelectric properties.

#### 3.2.5. Ferroelectric Hysteresis Measurements

**Figure 15:**
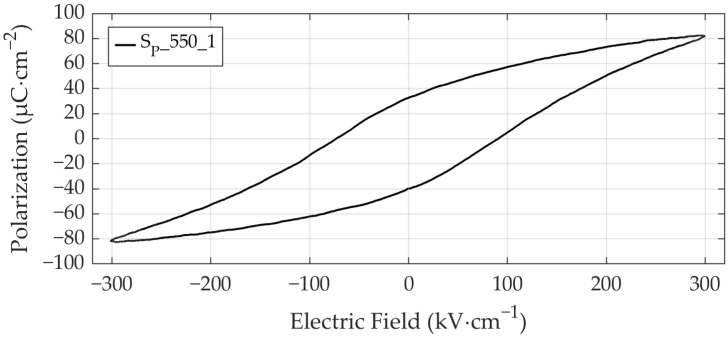
Ferroelectric hysteresis loop of sample S_P__550_1 exhibiting the highest saturation polarization (82.8 µC·cm^−2^) and remnant polarization (36.9 µC·cm^−2^) among all samples.

**Figure 16:**
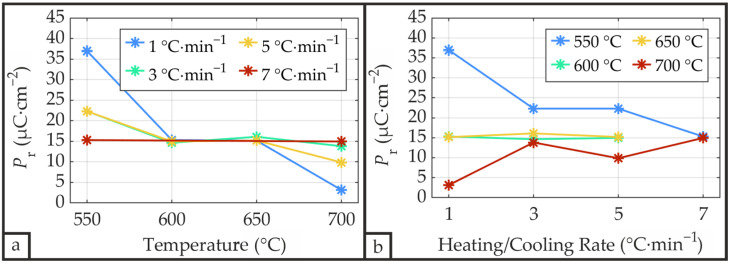
Remnant polarization *P*_r_ of the PZT thin films as a function of annealing temperature (**a**) and heating/cooling rate (**b**) measured using a Sawyer-Tower circuit at a maximum applied electric field of 300 kV·cm^−1^.

**Figure 17:**
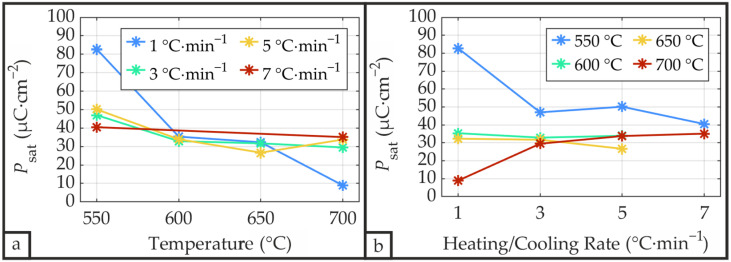
Saturation polarization *P*_sat_ of the PZT thin films as a function of annealing temperature (**a**) and heating/cooling rate (**b**) measured using a Sawyer-Tower circuit at a maximum applied electric field of 300 kV·cm^−1^.

Figure 15 presents the ferroelectric hysteresis loop of sample S_P__550_1 featuring the highest saturation polarization (82.8 µC·cm^−2^) and remnant polarization (36.9 µC·cm^−2^) among all samples. Wang and Zou [13], investigating different annealing temperatures, reported their highest remnant polarization of 27 µC·cm^−2^ for PZT annealed at a comparable temperature of 575 °C via CFA, using a similar fabrication process including sputtered Pb_1.1_Zr_0.52_Ti_0.48_O_3_ films with a thickness of 800 nm deposited on unheated Si/SiO_2_/Ti/Pt substrates. Remnant and saturation polarization data for all patterned samples are displayed in Figure 16 and Figure 17, with *P*_r_ and *P*_sat_ exhibiting a highly proportional relationship. Electrical characterization of samples S_P__600_7 and S_P__650_7 could not be performed due to short circuits caused by hillocks with cracks (see Figure 6). The average remnant polarization values of *P*_r_ = 14.96 µC·cm^−2^ and *P*_r_ = 15.47 µC·cm^−2^ for samples annealed at 600 °C and 650 °C, respectively, are in good agreement with reported values, including Teeramongkonrasmee et al. [25] (*P*_r_ = 15 µC·cm^−2^ at 600 °C) and Velu et al. [16] (*P*_r_ = 16 µC·cm^−2^ at 625 °C), where comparable fabrication parameters were employed.

The remnant polarization shows a pronounced annealing temperature dependence (see Figure 16a), particularly at low heating/cooling rates, with the highest *P*_r_ values occurring at 550 °C, while the lowest values occur at 700 °C. Consequently, *P*_r_ exhibits a decreasing trend with increasing heating/cooling rate at an annealing temperature of 550 °C and an increasing trend at 700 °C (Figure 16b). For temperatures of 600 °C and 650 °C, *P*_r_ shows no significant dependence on heating/cooling rate. The largest variation in *P*_r_ occurs at a heating/cooling rate of 1 °C·min^−1^, with this variation decreasing at higher rates, as *P*_r_ approaches an average value of approximately 15 µC·cm^−2^. This indicates that, within the investigated parameter range, the effect of peak annealing temperature on remnant polarization decreases as heating/cooling rates increase.

The unpatterned sample S_U__700_1, which corresponds to the patterned sample with the lowest remnant polarization (S_P__700_1, *P*_r_ = 3.1 µC·cm^−2^), also exhibits the smallest crystallite size determined by the Williamson-Hall method (Figure 12a,b) and among the lowest by the Scherrer method (Figure 11), suggesting a correlation between ferroelectric properties and crystallite size. Similarly, the combination that yields the largest remnant polarization (550 °C annealing temperature with 1 °C·min^−1^ heating/cooling rate) features one of the largest crystallite sizes by the Scherrer method. Additionally, both the Scherrer and Williamson-Hall methods reveal a decreasing crystallite size with increasing temperature at 1 °C·min^−1^, consistent with the remnant polarization behavior observed for this heating/cooling rate. Beyond these observations, no distinct correlation between crystallite size and remnant polarization could be identified. Zhu et al. [33] also demonstrated that ferroelectric properties cannot be reliably inferred from XRD patterns, emphasizing the necessity of direct ferroelectric measurements.

#### 3.2.6. Capacitance Measurements

**Figure 18:**
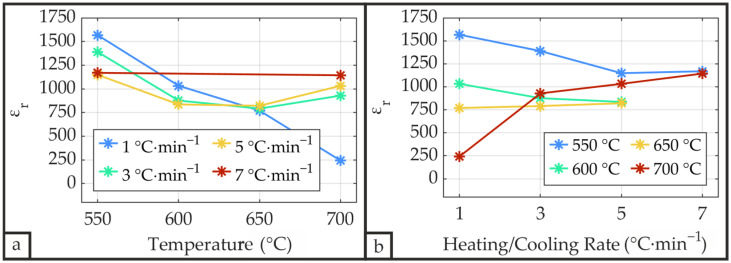
Relative permittivity *ε*_r_ of the PZT thin films as a function of annealing temperature (**a**) and heating/cooling rate (**b**) measured using an LCR meter at 10 kHz with an applied voltage of 1 V (8.77 kV·cm^−1^) and calculated from Equation (8).

Figure 18 presents the relative permittivity *ε*_r_ of the PZT thin films, calculated from capacitance measurements according to Equation (8). The relative permittivity shows a pronounced correlation with the ferroelectric properties *P*_r_ and *P*_sat_ (Figure 16 and Figure 17), consistent with findings reported by Teeramongkonrasmee et al. [25] and Haccart et al. [26]. The measured values (750–1200) fall within the typical range for PZT thin films with a thickness of approximately 1 µm [13,25,26,32]. The highest *ε*_r_ values are measured for samples annealed at low temperatures in combination with low heating/cooling rates. Although samples annealed at a rate of 1 °C·min^−1^ exhibit a distinct reduction in relative permittivity with increasing temperature, attributable to Pb volatilization driven by the elevated thermal budget, no pronounced temperature dependence is otherwise observed. Overall, the highest relative permittivity occurs at low heating/cooling rates combined with low annealing temperatures, with sample S_P__550_1 reaching a maximum *ε*_r_ = 1567.

#### 3.2.7. Leakage Current Measurements

**Figure 19:**
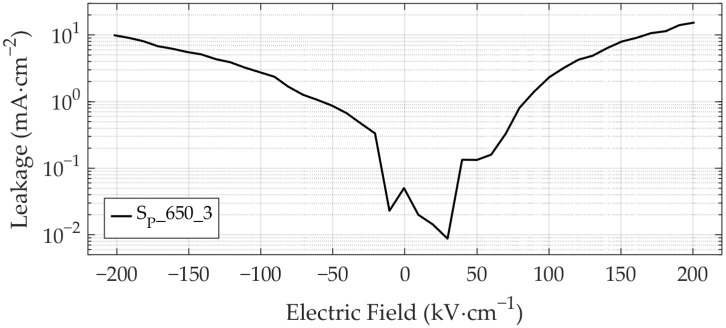
Leakage current density as a function of applied electric field is shown exemplarily for sample S_P__650_3.

**Figure 20:**
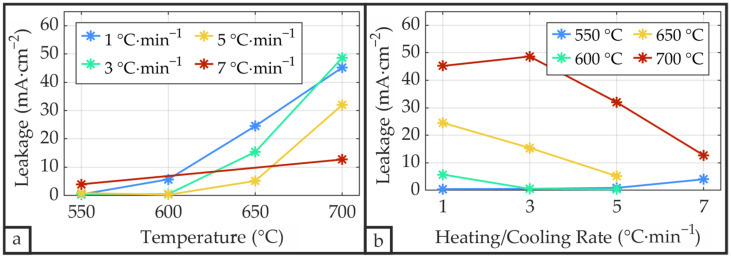
Maximum leakage current density of the PZT thin films measured at an applied electric field of up to ±200 kV·cm^−1^ as a function of annealing temperature (**a**) and heating/cooling rate (**b**).

Figure 19 presents the leakage current density as a function of applied electric field up to ±200 kV·cm^−1^ for the representative sample S_P__650_3. The leakage current features nearly symmetrical behavior, peaking at the highest applied electric fields and reaching a minimum of approximately 10^−2^ mA·cm^−2^ at zero bias, which represents the lowest detectable signal. Peak leakage current density values for all samples are displayed in Figure 20. The leakage current exhibits strong temperature dependence, increasing significantly with temperature (Figure 20a), consistent with observations by Singh et al. [12]. While leakage current density is minimal and rate-independent at low annealing temperatures, the heating/cooling rate has progressively greater influence at elevated temperatures. As it is evident from Figure 20b, the leakage current density shows a decreasing trend with increasing heating/cooling rate at annealing temperatures of 650 °C and 700 °C. Consequently, leakage current minimization can be achieved by either using low annealing temperatures or, if high annealing temperatures are necessary, by applying high heating/cooling rates.

## 4. Summary and Conclusions

In this study, we systematically investigated the influence of heating/cooling rate (1 °C·min^−1^–7 °C·min^−1^) and annealing temperature (450 °C–700 °C) on the structural, electrical, and ferroelectric properties of 1.14 µm thick PZT thin films sputter-deposited on Si/SiO_2_/Ti/Pt substrates and crystallized via CFA. XRD analyses revealed complete perovskite phase formation at temperatures of 500 °C and above, independent of the heating/cooling rate. Crystallite size exhibited greater sensitivity to heating/cooling rate than to the peak annealing temperature at temperatures below 700 °C. While microstrain increased significantly with higher heating/cooling rates and showed little dependence on annealing temperature, out-of-plane lattice strain *e*_⊥,(111)_ was predominantly affected by annealing temperature. An overview of the electrical properties and a comparison with the studies referenced in Table 1 are listed in Table 4.

Ferroelectric characterization showed that the highest remnant polarization (36.9 µC·cm^−2^) was achieved through low-temperature annealing (550 °C) combined with a slow heating/cooling rate (1 °C·min^−1^). The leakage current remained relatively low up to temperatures of 600 °C across all heating/cooling rates, but increased substantially above this threshold, particularly for low heating/cooling rates.

Additionally, we investigated the impact of substrate preparation on the quality of the PZT film. A comparison between PECVD and sputtered SiO_2_ layers demonstrated that high-quality SiO_2_ is essential to prevent hillock formation in the Ti/Pt bottom electrode that would otherwise affect the PZT layer. Furthermore, thermal stabilization of the Ti/Pt bottom electrode prior to PZT deposition proved to be crucial for improving the PZT film quality. This pre-treatment enhanced nucleation during subsequent PZT crystallization and effectively suppressed both hillock formation and delamination of the PZT thin film. However, films annealed at high heating/cooling rates (7 °C·min^−1^) remained susceptible to hillock formation accompanied by crack development, indicating stress-induced defect formation from rapid thermal cycling.

These findings demonstrate that precise control of both substrate preparation and thermal processing parameters, including the heating/cooling rate, is essential for optimizing PZT thin film properties. The best results were obtained using a thermally stabilized Ti/Pt bottom electrode on sputtered SiO_2_ in combination with slow heating/cooling rates at annealing temperatures of 550 °C–600 °C, balancing high remnant polarization with good surface morphology and low leakage current.

## Figures and Tables

**Table 1:** Summary of selected studies examining the annealing process of RF magnetron sputtered PZT near the morphotropic phase on Si/SiO_2_/Ti/Pt substrates and their annealing parameters evaluated.

Authors	PZT Deposition Temperature (°C)	Annealing Method	Annealing Temperature (°C)	Dwell Time	Heating Rate (°C·min^−1^)
Velu et al. [16]	unheated	CFA	500–625	30 min, 120 min	3
	unheated	RTA	550–700	5 s–120 s	420
Huang et al. [30]	unheated	CFA	510–600	10 min	20
	unheated	RTA	550–700	60 s	3000
Hu et al. [31]	unheated	CFA	450–700	0 min–80 min	10
	unheated	RTA	350–700	1 s–60 s	6000
Mardare et al. [22]	unheated	RTA	650	1 min, 10 min	60, 600
Chang and Chen [29]	350	RTA	650–850	5 min–80 min	N/A
Zhu et al. [33]	unheated	RTA	600–700	1 min	N/A
Thongrit et al. [10]	unheated	CFA	550–700	60 min	5
Wang and Zou [13]	N/A	CFA	525–625	10 min–40 min	N/A
Thomas et al. [32]	300	CFA	450–650	20 min–360 min	N/A

**Table 2:** Deposition parameters used for the sample preparation.

Parameter	SiO_2_	Ti	Pt	PZT
Sputtering Method	RF magnetron sputtering	DC magnetron sputtering	DC magnetron sputtering	RF magnetron sputtering
Target Material	Si	Ti	Pt	Pb(Zr_0.52_Ti_0.48_)O_3_
Target Diameter	2·100 mm	100 mm	100 mm	100 mm
Power	1000 W	400 W	400 W	400 W
Process Pressure	5 × 10^−3^ mbar	5 × 10^−3^ mbar	5 × 10^−3^ mbar	5 × 10^−3^ mbar
Sputtering Medium	Ar (95 sccm) O_2_ (5 sccm)	Ar (35 sccm)	Ar (35 sccm)	Ar (35 sccm)
Substrate Temperature	unheated	unheated	unheated	unheated
Deposition Rate	11.9 nm·min^−1^	6.2 nm·min^−1^	26.1 nm·min^−1^	8.2 nm·min^−1^
Film Thickness	300 nm	10 nm	170 nm	1.14 µm

**Table 3:** Sample identifiers for each combination of peak temperature and heating/cooling rate for patterned samples.

		Temperature			
		550 °C	600 °C	650 °C	700 °C
**Heating/Cooling Rate**	**1 °C·min^−1^**	S_P__550_1	S_P__600_1	S_P__650_1	S_P__700_1
	**3 °C·min^−1^**	S_P__550_3	S_P__600_3	S_P__650_3	S_P__700_3
	**5 °C·min^−1^**	S_P__550_5	S_P__600_5	S_P__650_5	S_P__700_5
	**7 °C·min^−1^**	S_P__550_7	S_P__600_7	S_P__650_7	S_P__700_7

**Table 4:** Reported electrical properties of selected studies examining the annealing process of RF magnetron sputtered PZT near the morphotropic phase on Si/SiO_2_/Ti/Pt.

Authors	PZT’s Thermal Treatment (Method, Temperature, Dwell Time, Heating Rate)	*P*_r_ (µC·cm^−2^)	*P*_max_ (µC·cm^−2^)	*ε*_r_	Leakage Current
This Study	CFA, 550 °C, 30 min, 3 °C·min^−1^	37 at 300 kV·cm^−1^ (25 at 200 kV·cm^−1^)	83 at 300 kV·cm^−1^ (59 at 200 kV·cm^−1^)	1567 at 10 kHz	0.4 mA·cm^−2^ at 200 kV·cm^−1^
Velu et al. [16]	CFA, 625 °C, 30 min, 3 °C·min^−1^	16 at 200 kV·cm^−1^	38 at 200 kV·cm^−1^	N/A	N/A
	RTA, 625 °C, 30 s, 420 °C·min^−1^	20 at 200 kV·cm^−1^	38 at 200 kV·cm^−1^	N/A	N/A
Huang et al. [30]	CFA, N/A, 10 min, 20 °C·min^−1^	5 at 250 kV·cm^−1^	10 at 250 kV·cm^−1^	N/A	N/A
	RTA, N/A, 60 s, 3000 °C·min^−1^	20 at 250 kV·cm^−1^	45 at 250 kV·cm^−1^	N/A	N/A
Mardare et al. [22]	RTA, 650 °C, 10 min, 60 °C·min^−1^	28 at 600 kV·cm^−1^	45 at 600 kV·cm^−1^	N/A	90 mA·cm^−2^ at 400 kV·cm^−1^
Chang and Chen [29]	RTA, 650 °C, 20 min, N/A	0.1 at N/A	N/A	869 at 1 kHz	N/A
Zhu et al. [33]	RTA, 650 °C, 1 min, N/A	28 at 520 kV·cm^−1^	43 at 520 kV·cm^−1^	N/A	N/A
Wang and Zou [13]	CFA, 575 °C, 30 min, N/A	27 at 200 kV·cm^−1^	40 at 200 kV·cm^−1^	950 at 1 kHz	N/A
Thomas et al. [32]	CFA, 550 °C, 45 min, N/A	18 at 200 kV·cm^−1^	40 at 200 kV·cm^−1^	992 at 10 kHz	N/A

## Data Availability

The original contributions presented in the study are included in the article; further inquiries can be directed to the corresponding author.

## Abbreviations

The following abbreviations are used in this manuscript:

*A*	Area
AFM	Atomic Force Microscopy
*β*	Full Width at Half Maximum
*C*	Capacitance
CFA	Conventional Furnace Annealing
CTE	Coefficient of Thermal Expansion
*d*	Thickness
*D*	Crystallite Size
*ε*	Microstrain
*ε*_0_	Vacuum Permittivity
*ε*_r_	Relative Permittivity
*e*_⊥__,(111)_	Out-of-Plane Lattice Strain Derived from the (111) Diffraction Peak
*E*_c_	Coercive Electric Field
FWHM	Full Width at Half Maximum
(*hkl*)	Plane
*I*	Intensity
*K*	Scherrer Constant
MEMS	Micro-Electromechanical Systems
*n*	Order
N/A	Not Applicable/Not Available
*ω*	Angle of Incident
O	Oxygen
*O*	Crystallographic Orientation
*P*_r_	Remnant Polarization
*P*_sat_	Saturation Polarization
Pb	Lead
PECVD	Plasma Enhanced Chemical Vapor Deposition
Pt	Platinum
PZT	Lead Zirconate Titanate
*R*_a_	Arithmetical Mean Roughness
*R*_q_	Root Mean Square Roughness
RF	Radio-Frequency
RTA	Rapid Thermal Annealing
S_P_	Patterned Sample
S_U_	Unpatterned Sample
SEM	Scanning Electron Microscopy
Si	Silicon
SiO_2_	Silicon Dioxide
*θ*	Bragg Angle
Ti	Titanium
XRD	X-Ray Diffraction
Zr	Zirconium
